# Correlation between endothelial function and carotid atherosclerosis in rheumatoid arthritis patients with long-standing disease

**DOI:** 10.1186/ar3382

**Published:** 2011-06-22

**Authors:** Carlos González-Juanatey, Javier Llorca, Miguel A González-Gay

**Affiliations:** 1 Cardiology Division, Hospital Xeral-Calde, c/Dr. Ochoa s/n, Lugo E-27004, Spain; 2 Department of Epidemiology and Computational Biology, School of Medicine, University of Cantabria, and CIBER Epidemiología y Salud Pública (CIBERESP), IFIMAV, Avenida Herrera Oria s/n, E-39011 Santander, Spain; 3 Department of Rheumatology, Hospital Universitario Marqués de Valdecilla, IFIMAV, Avenida de Valdecilla s/n, E-39008, Santander, Spain

## Abstract

**Introduction:**

In this study, we aimed to determine the relationship between flow-mediated endothelium-dependent vasodilatation (FMD) and carotid artery intima-media wall thickness (IMT), two surrogate markers of atherosclerosis, in a series of Spanish patients with rheumatoid arthritis (RA) without clinically evident cardiovascular (CV) disease.

**Methods:**

One hundred eighteen patients who fulfilled the 1987 American College of Rheumatology classification criteria for RA, had no history of CV disease and had at least one year of follow-up after disease diagnosis were randomly selected. Brachial and carotid ultrasonography were performed to determine FMD and carotid IMT, respectively.

**Results:**

Carotid IMT values were higher and FMD percentages derived by performing ultrasonography were lower in individuals with a long duration from the time of disease diagnosis. Patients with a disease duration ≤ 7 years had significantly lower carotid IMT (mean ± SD) 0.69 ± 0.17 mm than those with long disease duration (0.81 ± 0.12 mm in patients with ≥ 20 years of follow-up). Also, patients with a long disease duration had severe endothelial dysfunction (FMD 4.0 ± 4.0% in patients with disease duration from 14.5 to 19.7 years) compared with those with shorter disease duration (FMD 7.4 ± 3.8% in patients with disease duration ≤ 7 years). Linear regression analysis revealed that carotid IMT was unrelated to FMD in the whole sample of 118 patients. However, carotid IMT was negatively associated with FMD when the time from disease diagnosis ranged from 7.5 to 19.7 years (*P *= 0.02).

**Conclusions:**

In patients with RA without CV disease, endothelial dysfunction and carotid IMT increased with the duration of RA. The association between FMD and carotid IMT values was observed only in patients with long disease duration.

## Introduction

Rheumatoid arthritis (RA) is a chronic inflammatory disease associated with increased incidence of cardiovascular (CV) mortality [1,2]. This is the result of accelerated atherosclerosis [3]. Because of the high incidence of CV events observed in patients with RA, an important step forward might be to identify high-risk individuals who would benefit from active therapy to prevent clinical disease. In this regard, several noninvasive imaging techniques offer clinicians a unique opportunity to study the relationship of surrogate markers to the development of atherosclerosis. Among them, ultrasound techniques based on flow velocity and intimal thickness are considered efficient ways to measure subclinical atherosclerosis. Using brachial artery ultrasonography assessment, we and others have found the presence of endothelial dysfunction expressed by abnormal levels of flow-mediated endothelium-dependent vasodilatation (FMD) in patients without clinically evident CV disease who had either long-standing RA [4] or early-onset RA [5]. Moreover, increased carotid artery intima-media wall thickness (IMT) and increased frequency of carotid plaques have been described in RA patients with or without classic CV risk factors compared to ethnically matched controls [6-11]. Also, besides an association of carotid IMT with markers of inflammation [12,13], the duration of the disease has been associated with an increase in carotid IMT [14] and the presence of carotid plaques [8,11]. This is in accordance with data showing progression of carotid IMT in RA patients with long-standing, severe disease despite treatment with anti-TNF-α therapy [15]. Interestingly, a recent observation disclosed that carotid IMT may predict the development of CV events in patients with RA [16].

Since FMD constitutes a physiologic assessment of endothelial dysfunction and carotid IMT is an anatomic structural measure of subclinical atherosclerosis, it is logical that FMD might be a more useful diagnostic marker than carotid IMT in the early stages of the disease. In contrast, carotid IMT might be considered in the assessment of CV risk among patients with long-standing RA.

No relationship between carotid IMT and brachial artery FMD was found in middle-aged men without a history of CV disease who were considered to be at low or intermediate risk for future CV events based on current risk stratification algorithms [17]. However, because of the increased risk of CV events, this may not be the case for patients with long-standing RA.

Taking into consideration all of these factors together, and based on the experience of our group in the study of subclinical atherosclerosis not only in RA but also in other chronic inflammatory rheumatic diseases using ultrasound techniques [18-20], in the present study we aimed to determine the relationship between FMD and carotid IMT in RA patients without clinically evident CV disease.

## Materials and methods

### Patients and study protocol

Between January 2008 and December 2009, a series of 118 patients attending the rheumatology outpatient clinic of Hospital Xeral-Calde, Lugo, Spain, who fulfilled the 1987 American College of Rheumatology classification criteria for RA [21] and had no history of CV disease and but had at least one year of follow-up from their disease diagnosis were randomly selected for ultrasonographic assessment. To determine whether endothelial dysfunction was present, FMD was assessed by brachial ultrasonography as previously reported [18,22]. An FMD value < 7% was considered pathologic, indicating the presence of endothelial dysfunction [22]. Intraobserver variability for FMD and NTG was 1.3% and 1.9%, respectively, based on repeat brachial ultrasonography in 32 healthy controls. Assessment of the endothelial function of patients undergoing anti-TNF-α therapy was performed 24 to 48 hours before drug administration. Also, carotid ultrasonography was performed to determine carotid artery IMT. IMT was assessed in the right common carotid artery as previously reported [19,22]. On the basis of a second carotid ultrasonography performed in 20 RA patients and 20 healthy controls within one week after the first assessment, the correlation coefficient for carotid IMT was 0.98. The main epidemiological data of this series of patients are shown in Table 1. The patients' written consent was obtained according to the Declaration of Helsinki, and the design of the work was approved by the Ethics Committee of Galicia (Spain).

**Table 1:** Main epidemiologic data for 118 patients with RA who underwent ultrasonography^a^

Variable	Mean ± SD or number of patients (%)	Median (IQR)
Age at the time of the study, years	58.4 ± 12.9	59.5 (49 to 68.5)
Disease duration from RA diagnosis, years	13.8 ± 7.7	14 (7 to 19.7)
Women	89 (75.4%)	
Rheumatoid factor-positive	96 (81.4%)	

^a^RA: rheumatoid arthritis, SD: standard deviation, IQR: interquartile range.

### Statistical analysis

Quantitative variables are described using means and standard deviations (SDs) and medians and interquartile ranges (IQRs), and qualitative variables are described as numbers and percentages. The relationship between carotid IMT (as a dependent variable) and FMD (as an independent variable) was explored using linear regression, adjusting for gender, age at the time of RA diagnosis and years from RA disease diagnosis to ultrasonographic assessment. To further explore this relationship, we repeated the regression analysis by stratifying patients into quartiles defined by the time from RA diagnosis to ultrasonographic evaluation.

## Results

Patients with RA were stratified into four quartiles according to the time from disease diagnosis to ultrasonographic assessment (Table 2). Following this procedure, we observed that carotid IMT values were greater in individuals with a longer duration from disease diagnosis to ultrasonographic assessment (*P *< 0.001). In this regard, carotid IMT values were higher if time from RA diagnosis was longer than its median (that is, > 14 years). With respect to this observation, individuals with disease duration from RA diagnosis ≤ 7 years had significantly lower carotid IMT wall thickness (0.69 ± 0.17 mm) than did those with long disease duration (0.81 ± 0.12 mm in individuals with at least 20 years of follow-up from the time of RA diagnosis). Likewise, FMD decreased as time from RA diagnosis increased (*P *< 0.001). As shown in the carotid artery ultrasonographic evaluation, individuals with longer disease duration from disease diagnosis had severe endothelial dysfunction (FMD 4.0 ± 4.0% in RA patients with disease duration between 14.5 and 19.7 years versus 3.3 ± 4.4% in those with disease duration ≥ 20 years) compared with RA patients who had shorter disease duration (FMD 7.4 ± 3.8% in patients with disease duration ≤ 7 years).

**Table 2:** Distribution of carotid IMT and FMD in RA patients stratified into four quartiles according to time from disease diagnosis to ultrasonography^a^

Time from RA diagnosis to ultrasonography	Carotid IMT (mm) mean ± SD	FMD (%) mean ± SD
Quartile 1, one to seven years	0.69 ± 0.17	7.4 ± 3.8
Quartile 2, 7.5 to 14 years	0.68 ± 0.16	6.6 ± 4.6
Quartile 3, 14.5 to 19.7 years	0.84 ± 0.24	4.0 ± 4.0
Quartile 4, 20 to 38 years	0.81 ± 0.12	3.3 ± 4.4
*P *value	< 0.001	< 0.001

^a^Carotid IMT: carotid intima-media wall thickness, FMD: flow-mediated endothelium-dependent vasodilatation, RA: rheumatoid arthritis.

**Figure 1:**
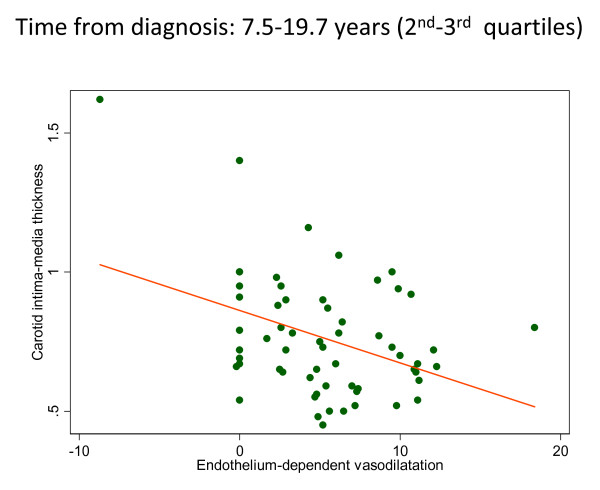
**Scatterplot illustrating the relationships between FMD and carotid IMT in patients with RA and disease duration ranging between 7 and 20 years derived using linear regression analysis**.

To explore the relationship between FMD and carotid IMT, linear regression analysis was performed. This analysis disclosed that carotid IMT was unrelated to FMD endothelium-dependent vasodilatation in the whole sample of 118 patients (Table 3). However, when patients with RA were stratified according to the time from disease diagnosis until the time of ultrasonography, carotid IMT was negatively associated with FMD when the time from disease diagnosis ranged from 7.5 to 19.7 years (*P *= 0.02). In patients included in this range of disease duration, the higher FMD percentages were associated with thinner (that is, lower) carotid IMT values (Figure 1). However, in patients with shorter disease duration (one to seven years) or longer disease duration (≥ 20 years), FMD and carotid IMT values remained unrelated (Table 4).

**Table 3:** Regression analysis between carotid IMT and FMD^a^

Variable	Correlation coefficient (95% confidence interval)	*P *value
FMD^b^	-0.003 (-0.009 to 0.003)	0.35

		

^a^Carotid IMT: carotid intima-media wall thickness, FMD: flow-mediated endothelium-dependent vasodilatation, RA: rheumatoid arthritis; ^b^Adjusted for gender, age at the time of disease diagnosis and years from RA diagnosis to ultrasonography assessment.

**Table 4:** Regression analysis between carotid IMT and FMD stratified by disease duration from the time of RA diagnosis until ultrasonography assessment^a^

Time from RA diagnosis until ultrasonography	Correlation coefficient (95% confidence interval)	*P *value
Quartile 1, one to seven years	-0.003 (-0.019 to 0.013)	0.82
Quartiles 2 and 3, 7.5 to 19.7 years	-0.012 (-0.021 to -0.003)	0.02
Quartile 4, 20 to 38 years	0.008 (-0.003 to 0.019)	0.27

^a^Carotid IMT: carotid intima-media wall thickness, FMD: flow-mediated endothelium-dependent vasodilatation, RA: rheumatoid arthritis. Data are adjusted for gender, age at time of diseaseand time from RA diagnosis until ultrasonography.

## Discussion

A series of cellular, molecular and pathophysiological events occur during the progression of RA and may also be involved first in endothelial dysfunction and later in atherosclerosis [23]. A healthy endothelium prevents adhesion of mononuclear cells. Inflammation promotes endothelial cell activation, which is characterized by loss of vascular integrity, increased expression of leukocyte adhesion molecules such as selectins, vascular cell adhesion molecule 1 and intercellular adhesion molecule 1 (ICAM-1); change in phenotype from antithrombotic to thrombotic; production of several cytokines; and upregulation of human leukocyte antigen molecules. All of these changes allow endothelial cells to participate in the inflammatory response. In this process, the increased expression of adhesion molecules promotes the adherence and migration of monocytes into the vessel wall. Differentiation of monocytes into macrophages in the intima and activation and further differentiation to form cells characterize the development of early atherosclerotic lesions [24,25]. Continuous endothelial cell activation, manifested by increased levels of the adhesion molecules soluble ICAM-1 and sE-selectin, is present in patients with RA [26]. This endothelial cell activation subsequently leads to endothelial dysfunction, which is an important event in early atherogenesis and also contributes to the development of clinical features in the later stages of the vascular disease, including the progression of atherosclerotic plaque [27]. Interestingly, Kerekes *et al*. [28] showed that disease duration was associated with impaired FMD and several biomarkers of inflammation. Although in the present study we found no impairment of endothelial function as determined on the basis of brachial FMD in patients with < 7 years' disease duration, longer disease duration, particularly > 14 years, was associated with severe endothelial dysfunction. In keeping with this observation, Södergren *et al*. [29] found no impairment in endothelial function, measured as FMD, among patients with newly diagnosed RA compared with controls.

With regard to carotid ultrasonography, it is known that a common carotid artery IMT ≥ 0.60 mm is a marker of atherosclerosis [30,31]. In addition, both carotid artery IMT > 0.90 mm and the presence of carotid plaques are considered to be expression of subclinical organ damage and factors influencing CV prognosis in the general population [32]. Interestingly, a recent meta-analysis demonstrated an increased carotid IMT in patients with early RA [33]. Although the increase in carotid IMT in this meta-analysis was much lower than expected in view of the almost doubled CV risk in RA patients [33], carotid IMT was proven to predict the development of CV events in RA patients [16]. Because of that finding, the presence of abnormally high carotid IMT values should be raise clinical suspicions as a sign of the development of CV complications in these patients. In the present study, we observed that individuals with long disease duration (> 14 years) had abnormally high carotid IMT values. With respect to this observation, on the basis of 631 consecutive RA patients, del Rincón *et al*. [14] showed that IMT increases per unit of age in proportion to RA duration. In del Rincón *et al*.'s study [14], carotid IMT increased from 0.154 mm/10 years among patients with RA for ≤ 7 years to 0.295 mm/10 years among patients with RA for ≥ 20 years.

In line with the above findings, using electron-beam computed tomography to measure the extent of coronary artery calcification, Chung *et al*. [34] found that coronary-artery calcification occurred more frequently in patients with established RA than in patients with early RA and controls. A question that needs to be answered is whether carotid ultrasonography and brachial FMD should routinely be performed in all patients with RA to improve CV risk management. With respect to this question, carotid IMT was found to be an independent predictor of vascular events in high-risk individuals without RA in whom risk factors were managed clinically [35]. Since the risk of CV disease is increased in patients with RA, carotid ultrasound might be a potential additional tool for stratifying CV risk in patients with RA [22]. In this regard, a recent study by Evans *et al*. [36] showed that the presence of carotid plaques in both internal carotid arteries following carotid ultrasonography nearly quadrupled the incidence of new acute coronary syndromes in patients with RA compared with those in RA patients without carotid plaques. On the hand, impaired FMD of the brachial artery due to endothelial dysfunction has been associated with both CV risk factors and future CV morbidity and mortality in the general population [37]. In addition, endothelial dysfunction manifested by impaired FMD was observed in both long-standing RA patients [4] and early-onset RA patients [5] without clinically evident CV disease. These observations support a potential role of FMD in establishing the presence of endothelial dysfunction as a subclinical marker of atherosclerotic disease in RA. They may also provide a basis for the association between RA and atherosclerotic disease.

Of main clinical relevance may be the improvement in endothelial function observed in patients with RA following treatment with TNF-α blockers [38-40] or rituximab [41,42]. However, the beneficial effect of the TNF-α antagonist infliximab on endothelial dysfunction seems to be only temporary [43]. Conflicting results have been described regarding the effects of biologic agents on carotid atherosclerosis [44]. In this regard, while some patients showed significant improvement in carotid IMT following TNF-α blocker therapy [45], others did not experience reduction of IMT following treatment with these drugs [15,40,46].

Previous observations showed no relationship between carotid IMT and brachial artery FMD in middle-aged men at low and intermediate risk of experiencing future CV events [17]. However, the situation might not be the same in patients with long-standing RA. Therefore, we aimed to determine the relationship between both techniques in patients with RA. On the basis of linear regression analysis, we found that carotid IMT was unrelated to FMD in the whole sample of 118 patients. This observation was also in keeping with another study performed in elderly individuals that showed no correlation between brachial FMD and carotid IMT [47]. However, our data suggest that in patients with disease duration ranging between from 7.5 to 19.7 years, carotid IMT is negatively associated with brachial FMD.

## Conclusions

In summary, our results reinforce the importance of disease duration in the development of atherosclerosis in patients with RA. Brachial FMD and carotid IMT may indicate distinct and independent stages in the complex pathways leading to accelerated atherosclerosis in patients with RA.

## Abbreviations

CV: cardiovascular; IMT: intima-media thickness; IQR: interquartile range; FMD: endothelium-dependent flow-mediated vasodilatation; RA: rheumatoid arthritis; SD: standard deviation.

## Competing interests

The authors declare that they have no competing interests.

## Authors' contributions

CGJ performed the ultrasonographic studies, participated in the design of the study and helped to draft the manuscript. JL participated in the design of the study and data analysis and helped to draft the manuscript. MAGG made substantial contributions to the conception and design of the study, the acquisition of data and the coordination of the study. All authors read and approved the final version of the manuscript to be published.
